# Source Localization in Acoustic Sensor Networks via Constrained Least-Squares Optimization Using AOA and GROA Measurements

**DOI:** 10.3390/s18040937

**Published:** 2018-03-22

**Authors:** Ji-An Luo, Si-Wei Pan, Dong-Liang Peng, Zhi Wang, Yan-Jun Li

**Affiliations:** 1 Key Lab for IOT and Information Fusion Technology of Zhejiang, Hangzhou Dianzi University, Hangzhou 310018, China; 151060011@hdu.edu.cn (S.-W.P.); dlpeng@hdu.edu.cn (D.-L.P.); 2 The State Key Laboratory of Industrial Control Technology, Zhejiang University, Hangzhou 310027, China; wangzhizju@gmail.com; 3 School of Computer Science and Technology, Zhejiang University of Technology, Hangzhou 310023, China; yjli@zjut.edu.cn

**Keywords:** wireless sensor networks, source localization, sensor position uncertainty, angle of arrival, gain ratio of arrival

## Abstract

A constrained least-squares (CLS) 3D source localization method is presented for acoustic sensor networks with sensor position errors. The proposed approach uses angles of arrivals (AOAs) and gain ratios of arrival (GROAs) measured simultaneously at each node to estimate the source position jointly. Compared to AOA-only localization methods, the GROAs can be used in conjunction with AOA measurements so as to get more accurate results by exploiting the geometrical relationship between these two measurements. Compared to time difference of arrival localization methods, the proposed algorithm does not require accurate time synchronization over different nodes. The theoretical mean-square error matrices of the proposed approach are derived and they are exactly equal to the Cramér–Rao bound for Gaussian noise under the small error condition. Simulations validate the performance of the proposed estimator.

## 1. Introduction

Source localization is one of the key tasks for acoustic sensor networks (ASNs) [1,2,3]. It is typically required for surveillance or monitoring the environment, e.g., vehicle localization [1,2], helicopter localization [4] and animal localization [5,6]. In particular, when the spatially distributed nodes are equipped with acoustic arrays, two types of measurements, such as angle of arrival (AOA) [7] and gain ratio of arrival (GROA) [8], can be obtained locally using array signal processing techniques. Moreover, time difference of arrival (TDOA) [9,10] can also be obtained either in the centralized or distributed way after precise synchronization among the arrays. In fact, the above-mentioned metrics are complementary in terms of their geometry properties [11]. The usage of their combinations can improve the positioning accuracy [11,12,13,14,15].

In the ASN setting, acoustic arrays are randomly deployed in an area. Each array or node consists of several microphones for signal collection, a battery for energy supplies, a microprocessor for local computation and a radio for data communication. Due to the resource limitation, e.g., power supplement, wireless bandwidth, local computational capacity, etc., the localization task is generally divided into two steps [1]: (1) the AOA of the source signal is estimated in each local array; (2) those bearings are intersected to localize the target. In fact, the gain measurements can be acquired during the process of bearing estimation [16,17]. The additional gain ratio information can be utilized in conjunction with AOA measurements to improve source localization accuracy. In this paper, we solve the source localization problem using the AOA and GROA measurements jointly.

For AOA-only localization in ASNs, the target position can be estimated by intersecting bearing lines obtained from direction finding sensors at distinct locations. The highly nonlinear relationship between the source position and AOA measurements makes the localization problem nontrivial. For instance, the maximum likelihood (ML) cost functions using AOAs [18] are nonlinear and nonconvex under the Gaussian noise assumption. The iterative algorithms similar to Gauss–Newton [19] are commonly used to handle the nonlinearity with an initial guess. However, the global convergence of those iterative algorithms can not be guaranteed. To overcome the shortcomings of the ML method, a semi-definite relaxation [20] and a geometrical constrained optimization [21] are proposed to avoid the divergence problem of the ML approach for poor sensor-target geometry cases. However, those optimization algorithms are generally computing expensive, which obstructs their applications in sensor networks whose sensor nodes have limited computational capability. For this reason, algebraic solutions are also received much attention, including weighted least-squares (WLS) [14], constrained least-squares (CLS) [22] and weighted instrumental variable (WIV) [23,24] methods, etc. Basically, the AOA algebraic localization algorithms are mainly developed in two-dimensional (2D) space. Only a few papers have focused on the 3D scenario in formal literature. The authors in [23] firstly derived the closed-form bearing-only pseudolinear estimator (BOPLE) using the azimuth and elevation angles jointly. Although the BOPLE estimator is simple to implement, it has bias that does not vanish as the number of measurements increases. In [25], the authors pointed out that coordinate system rotation can be used to reduce the estimation bias. In [24], a two-stage weighted instrumental variable estimator has been developed and the estimation bias is compensated. In [26], the authors developed a bias reduced method for 3D AOA localization in wireless sensor networks with sensor position uncertainty. The bias is mainly formed by the correlation between the measurement matrix and the measurement vector. Compared to the WLS method, the bias can be reduced by the CLS algorithm.

When the signals are captured at the nodes, both bearing and signal amplitude information can be gathered. The signal strength can also be utilized to improve the localization accuracy. The motivation comes from the fact that the received acoustic signal intensity is inversely proportional to the distance between the source and the working node. As referred to in [27], one of the challenges for energy-based source localization is the nonconvex property of the cost function formulated from the maximum likelihood method [2]. The approach proposed in [27] applies a projection-onto-convex-sets method to form a convex feasibility problem, while Ref. [28] presented a semidefinite relaxation method to avoid plunging into local minima. The above methods focus on the nonlinear least-squares problem. Alternatively, Ref. [8] developed a closed-form solution using energy ratio measurements.

In this paper, we present a novel closed-form estimator for 3D source localization in ASNs based on hybrid AOA and GROA measurements when the node positions have errors. The proposed method assumes homogeneous atmospheric propagations, which have been commonly used in the related literature mentioned above. First, we linearize the nonlinear equations under the small Gaussian noise condition. We then derive an accurate closed-form estimator by utilizing new geometrical relationships between the hybrid measurements and the unknown source position. The proposed estimator can be implemented with a WLS method for simplicity if the number of sensors is small. For a large number of sensors, the estimator is realized by using a CLS algorithm to reduce the bias. Performance analysis shows that the theoretical mean-square error (MSE) of the proposed estimator can achieve the Cramér–Rao lower bound (CRLB) accuracy when the measurement errors are small. In summary, the main contributions can be listed as follows: (i) by utilizing the AOAs in conjunction with GROAs, we develop a new WLS estimator for 3D source localization; (ii) analogous to the bias reduction technique in [26], we present a CLS method to reduce the estimation bias with sensor position uncertainty; and (iii) we have proved that the theoretical MSE is equivalent to CRLB under a sufficiently small noise region.

The remainder of this paper is organized as follows. Section 2 provides the 3D hybrid measurement model. A Hybrid WLS algorithm is presented in Section 3. Section 4 derives the hybrid bias reduced estimator using a CLS approach. Simulation results are included in Section 5, and Section 6 contains conclusions.

## 2. Problem Formulation

**Figure 1:**
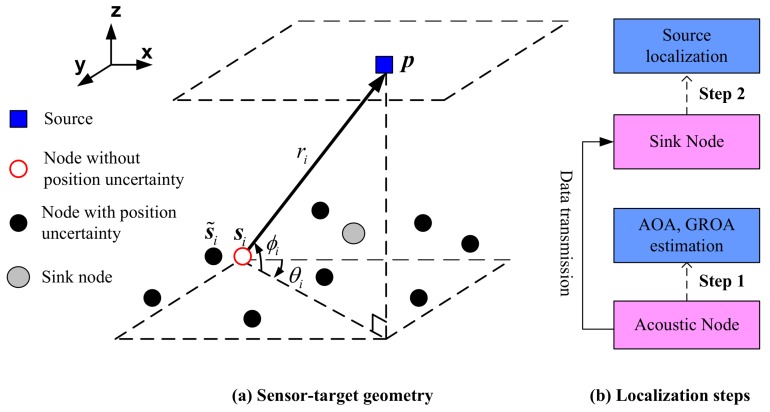
An example of 3D source localization for acoustic sensor networks.

We consider a 3D source localization problem in an ASN. Figure 1 shows a typical configuration of the ASN. *M* nodes are randomly dispersed at positions $s_{i}={\left[x_{i},y_{i},z_{i}\right]}^{T}$ and ${\left(\cdot \right)}^{T}$ is the transpose operation, i=1,…,M. The source locates at $p={\left[x,y,z\right]}^{T}$. In general, the localization task includes two steps: the first step is to measure AOAs and GROAs in acoustic nodes by exploiting the signals emitting from the target. As for step two, those measurements are communicated to the sink node where the source localization task is accomplished (see Figure 1b). Data transmission utilizes cluster tree topology, which contains sink node, cluster heads and local nodes. The sink node broadcasts network forming messages to nearby cluster heads, and these control messages are further transmitted to local nodes. As depicted in Figure 1a, an AOA measurement consists of the azimuth and elevation angles. The true azimuth angle $\theta_{i}$ and elevation angle $\phi_{i}$ are related to the source position and node *i* by

(1) $$\begin{array}{cc} \theta_{i} & =\mathrm{atan}2\left(\Delta y_{i},\Delta x_{i}\right), \end{array}$$

(2) $$\begin{array}{cc} \phi_{i} & =\mathrm{atan}2\left(\Delta z_{i},\Delta x_{i}\cos\theta_{i}+\Delta y_{i}\sin\theta_{i}\right), \end{array}$$

where $\Delta x_{i}=x-x_{i}$, $\Delta y_{i}=y-y_{i}$, $\Delta z_{i}=z-z_{i}$, $\theta_{i}\in \left(-\pi ,\pi \right)$ and $\phi_{i}\in \left(-\pi /2,\pi /2\right)$. Let $r_{i}={\left[\Delta x_{i},\Delta y_{i},\Delta z_{i}\right]}^{T}$ denote the range vector connecting sensor $s_{i}$ to the target p, and $r_{i}$ can be expressed as

(3) $$\begin{array}{c} r_{i}=p-s_{i}=r_{i}b_{i},\;i=1,\ldots ,M, \end{array}$$

where $r_{i}=∥p-s_{i}∥$ is the distance between the source and sensor *i*, ∥·∥ is the Euclidean norm, and $b_{i}={\left[\cos\theta_{i}\cos\phi_{i},\sin\theta_{i}\cos\phi_{i},\sin\phi_{i}\right]}^{T}$ is the normalized range vector. Similar to [12], we assume that the attenuation of signal gain is proportional to $r_{i}$ and the propagation medium is homogeneous. The true GROA received at sensor *j* with respect to the reference sensor 1 is

(4) $$\begin{array}{c} g_{j1}=r_{j}/r_{1}, \end{array}$$

where j=2,…,M, $r_{j}$ and $r_{1}$ represent the norm of $r_{j}$ and $r_{1}$, respectively.

In practice, the sensor measurements are affected by the additive noise and models (1), (2) and (4) become

(5) $$\begin{array}{c} {\tilde{\theta }}_{i}=\theta_{i}+n_{i},\;{\tilde{\phi }}_{i}=\phi_{i}+w_{i},\;{\tilde{g}}_{j1}=g_{j1}+e_{j}, \end{array}$$

respectively, where $n_{i}$, $w_{i}$ and $e_{j}$ are zero mean Gaussian noise terms. It should be noted that the Gaussian-noise assumption presented in Equation (5) is only applicable to specific scenarios when acoustic signals propagate through homogeneous atmosphere. In practice, this assumption might be invalid. For example, the performance of acoustic localization systems deployed in the atmosphere depends on atmospheric conditions. In most cases, atmospheric turbulence can not be neglected [29]. In addition, multi-path effects should also be considered [30], especially for urban or indoor scenarios. Due to these reasons, the additive noise is non-Gaussian and/or impulsive [31]. Nevertheless, Equation (5) provides a reasonable model that has been used by other researchers. The AOA model can be found in [24,26], and the GROA model can be found in [8,12]. The Gaussian noise assumption is a good starting point for us to study the localization performance by jointly utilizing the AOA and GROA measurements.

Moreover, we assume that the sensor positions also have errors. Let ${\tilde{s}}_{i}$ be the noisy sensor position vector. ${\tilde{s}}_{i}$ can be written as

(6) $$\begin{array}{c} {\tilde{s}}_{i}=s_{i}+\Delta s_{i}, \end{array}$$

where $\Delta s_{i}$ is the corresponding sensor position error. For practical applications, the node positions are often determined by self-localization [32] or GPS and the accuracy can not be perfect. Although the system error or bias may exist during self-positioning, it can be removed by sensor registration [33]. Similar to the assumption used in [26], we assume that $\Delta s_{i}$ follows zero mean Gaussian distribution. Being corrupted by noise, the measurement model can be written in vector form

(7) $$\begin{array}{c} \tilde{\chi }=\chi +\eta , \end{array}$$

where $\tilde{\chi }={\left[{\tilde{\theta }}^{T},{\tilde{\phi }}^{T},{\tilde{g}}^{T},{\tilde{s}}^{T}\right]}^{T}$ is the measurement vector, $\tilde{\theta }={\left[{\tilde{\theta }}_{1},{\tilde{\theta }}_{2},\ldots ,{\tilde{\theta }}_{M}\right]}^{T}$, $\tilde{\phi }={\left[{\tilde{\phi }}_{1},{\tilde{\phi }}_{2},\ldots ,{\tilde{\phi }}_{M}\right]}^{T}$ and $\tilde{g}={\left[{\tilde{g}}_{21},{\tilde{g}}_{31},\ldots ,{\tilde{g}}_{M1}\right]}^{T}$ are the collections of the AOA and GROA measurements, and $\tilde{s}={\left[{\tilde{s}}_{1}^{T},{\tilde{s}}_{2}^{T},\ldots ,{\tilde{s}}_{M}^{T}\right]}^{T}$ is the sensor position vector. $\chi ={\left[\theta^{T},\phi^{T},g^{T},s^{T}\right]}^{T}$, $\theta ={\left[\theta_{1},\theta_{2},\ldots ,\theta_{M}\right]}^{T}$, $\phi ={\left[\phi_{1},\phi_{2},\ldots ,\phi_{M}\right]}^{T}$ and $g={\left[g_{21},g_{31},\ldots ,g_{M1}\right]}^{T}$ are the vectors where the elements are the true values of AOA and GROA. and $s={\left[s_{1}^{T},s_{2}^{T},\ldots ,s_{M}^{T}\right]}^{T}$ is the true sensor position vector. $\eta ={\left[n^{T},w^{T},e^{T},\Delta s^{T}\right]}^{T}$ is the independent and identically distributed zero mean Gaussian noise with covariance matrix Q, where $n={\left[n_{1},n_{2},\ldots ,n_{M}\right]}^{T}$ is the azimuth angle measurement noise vector with covariance matrix $Q_{n}$, $w={\left[w_{1},w_{2},\ldots ,w_{M}\right]}^{T}$ is the elevation angle measurement noise vector with covariance matrix $Q_{w}$, $e={\left[e_{2},e_{3},\ldots ,e_{M}\right]}^{T}$ is the GROA measurement noise vector with covariance matrix $Q_{e}$, and $\Delta s={\left[\Delta s_{1}^{T},\Delta s_{2}^{T},\ldots ,\Delta s_{M}^{T}\right]}^{T}$ is the sensor position error vector with covariance matrix $Q_{s}$.

The objective of source localization problem is to estimate the location p as accurate as possible with all the available measurements, including azimuth angles $\tilde{\theta }$, elevation angles $\tilde{\phi }$ and GROA $\tilde{g}$.

## 3. WLS Estimator Using Joint AOA-GROA Measurements

Given measurement model (4), our task is to find the estimate of p that can attain the CRLB accuracy. Under the Gaussian noise assumption, it is certain that the ML estimation is optimal by minimizing the weighted MSE of χ. However, the ML cost function is nonlinear and nonconvex with respect to p. Numerical search is required to solve the ML nonlinear optimization problem, which is costly, and, therefore, we seek to establish a simple closed-form solution for estimating p using AOAs and GROAs jointly.

Let us define

(8) $$\begin{array}{cc} a_{\theta ,i} & ={\left[\sin\theta_{i},-\cos\theta_{i},0\right]}^{T},\\ a_{\phi ,i} & ={\left[\cos\theta_{i}\sin\phi_{i},\sin\theta_{i}\sin\phi_{i},-\cos\phi_{i}\right]}^{T}. \end{array}$$

Both $a_{\theta ,i}$ and $a_{\phi ,i}$ are orthogonal to $b_{i}$. Premultiplying Equation (3) with $a_{\theta ,i}$ and $a_{\phi ,i}$, Equation (3) can be rewritten as

(9) $$\begin{array}{c} F_{i}p-F_{i}s_{i}=0, \end{array}$$

where $F_{i}={\left[a_{\theta ,i},a_{\phi ,i}\right]}^{T}$. The relationships in Formula (9) is firstly derived in [23] using orthogonal vectors. The subspace method can facilitate the analysis for the source localization problem.

For GROAs, we first obtain the following equation from Equation (3)

(10) $$\begin{array}{c} 2p=s_{1}+s_{j}-\left(r_{j}-r_{1}\right)b_{1}+r_{j}\left(b_{j}+b_{1}\right). \end{array}$$

Premultiplying Formula (10) with ${\left(b_{j}-b_{1}\right)}^{T}$ yields

(11) $$\begin{array}{c} 2{\left(b_{j}-b_{1}\right)}^{T}p={\left(b_{j}-b_{1}\right)}^{T}\left[s_{1}+s_{j}-\left(r_{j}-r_{1}\right)b_{1}\right]. \end{array}$$

To derive Equation (11), the relationship $r_{j}{\left(b_{j}-b_{1}\right)}^{T}\left(b_{j}+b_{1}\right)=0$ is used, which was mentioned previously in [14]. Substituting the relations $r_{j}=g_{j1}r_{1}$ and $r_{1}b_{1}=p-s_{1}$ into (8), we have

(12) $$\begin{array}{c} \left(1+g_{j1}\right){\left(b_{j}-b_{1}\right)}^{T}p={\left(b_{j}-b_{1}\right)}^{T}\left(s_{j}+g_{j1}s_{1}\right). \end{array}$$

Putting the 2M equations for i=1,…,M from Equation (9) and the other M−1 equations for j=2,…,M from Formula (12) together yields the matrix form

(13) $$\begin{array}{c} Ap=h, \end{array}$$

where A is the measurement matrix and h is the measurement vector, $A={\left[A_{\theta }^{T},A_{\phi }^{T},V_{g}^{T}\right]}^{T}$, $A_{\theta }={\left[a_{\theta ,1},a_{\theta ,2},\ldots ,a_{\theta ,M}\right]}^{T}$, $A_{\phi }={\left[a_{\phi ,1},a_{\phi ,2},\ldots ,a_{\phi ,M}\right]}^{T}$, $V_{g}={\left[v_{2},v_{3},\ldots ,v_{M}\right]}^{T}$, $v_{j}=\left(1+g_{j1}\right)\left(b_{j}-b_{1}\right)$, $h={\left[h_{\theta }^{T},h_{\phi }^{T},u_{g}^{T}\right]}^{T}$, $h_{\theta }={\left[a_{\theta ,1}^{T}s_{1},a_{\theta ,2}^{T}s_{2},\ldots ,a_{\theta ,M}^{T}s_{M}\right]}^{T}$, $h_{\phi }={\left[a_{\phi ,1}^{T}s_{1},a_{\phi ,2}^{T}s_{2},\ldots ,a_{\phi ,M}^{T}s_{M}\right]}^{T}$, $u_{g}={\left[u_{2},u_{3},\ldots ,u_{M}\right]}^{T}$, and $u_{j}={\left(b_{j}-b_{1}\right)}^{T}\left(s_{j}+g_{j1}s_{1}\right)$.

In practice, only noisy vector $\tilde{\chi }$ is available. By employing these noisy measurements, Equation (13) becomes

(14) $$\begin{array}{c} \tilde{\eta }=\tilde{h}-\tilde{A}p, \end{array}$$

where $\tilde{\eta }$ denotes the pseudo-linear residual, $\tilde{A}$ and $\tilde{h}$ on the right side of Equation (14) are A and h with their actual values replaced by the measurements. When the measurement noise is small, $\sin n_{i}\approx n_{i}$, $\sin w_{i}\approx w_{i}$, $\cos n_{i}\approx 1$ and $\cos w_{i}\approx 1$, then we have the following approximations [26]

(15) $$\begin{array}{cc} \sin{\hat{\theta }}_{i} & \approx \sin\theta_{i}+n_{i}\cos\theta_{i},\;\cos{\hat{\theta }}_{i}\approx \cos\theta_{i}-n_{i}\sin\theta_{i},\\ \sin{\hat{\phi }}_{i} & \approx \sin\phi_{i}+w_{i}\cos\phi_{i},\;\cos{\hat{\phi }}_{i}\approx \cos\phi_{i}-w_{i}\sin\phi_{i}. \end{array}$$

According to Equations (9) and (15) and ${\tilde{s}}_{i}=s_{i}+\Delta s_{i}$, we obtain the noisy pseudo-linear equations of AOA measurements by neglecting second-order error terms

(16) $$\begin{array}{cc} {\tilde{a}}_{\theta ,i}^{T}{\tilde{s}}_{i}-{\tilde{a}}_{\theta ,i}^{T}p & \approx -n_{i}r_{i}\cos\phi_{i}+a_{\theta ,i}^{T}\Delta s_{i}, \end{array}$$

(17) $$\begin{array}{cc} {\tilde{a}}_{\phi ,i}^{T}{\tilde{s}}_{i}-{\tilde{a}}_{\phi ,i}^{T}p & \approx -w_{i}r_{i}+a_{\phi ,i}^{T}\Delta s_{i}. \end{array}$$

We then derive the noisy pseudo-linear equations for GROA measurements. The ranges from the target to sensors varies due to the sensor position uncertainty, and it leads to the change of the corresponding GROAs. With regard to noisy ranges, we have the following approximation when the sensor position error is small

(18) $$\begin{array}{c} {\tilde{r}}_{j}=r_{j}+b_{j}^{T}\Delta s_{j}. \end{array}$$

Accordingly, the noisy GROAs can be approximated by

(19) $$\begin{array}{c} {\tilde{g}}_{j1}\approx g_{j1}+\frac{b_{j}^{T}\Delta s_{j}}{r_{1}}+e_{j}. \end{array}$$

Using Equations (12), (15) and (19) and ${\tilde{s}}_{i}=s_{i}+\Delta s_{i}$, the noisy GROA pseudo-linear equation is given by

(20) $$\begin{array}{cc} {\tilde{u}}_{j}-{\tilde{v}}_{j}^{T}p\approx n_{1}r_{j}f_{n,1}^{T}b_{j}-n_{j}r_{j}f_{n,j}^{T}b_{1} & +w_{1}r_{j}f_{w,1}^{T}b_{j}-w_{j}r_{j}f_{w,j}^{T}b_{1}\\ & -e_{j}r_{1}{\left(b_{j}-b_{1}\right)}^{T}b_{1}+\Delta s_{1}g_{j1}{\left(b_{j}-b_{1}\right)}^{T}+\Delta s_{j}f_{s,j}^{T}, \end{array}$$

where $f_{n,i}={\left[-\sin\theta_{i}\cos\phi_{i},\cos\theta_{i}\cos\phi_{i},0\right]}^{T}$, $f_{w,i}={\left[-\cos\theta_{i}\sin\phi_{i},-\sin\theta_{i}\sin\phi_{i},\cos\phi_{i}\right]}^{T}$ and $f_{s,j}=\left(b_{j}-b_{1}\right)-b_{j}b_{1}^{T}\left(b_{j}-b_{1}\right)$. Substituting Formulas (16), (17) and (20) into Equation (14), we obtain

(21) $$\begin{array}{c} \tilde{\eta }=\tilde{h}-\tilde{A}p\approx G\eta , \end{array}$$

where G on the right side of Equation (21) is

(22) $$\begin{array}{c} G=\left[\begin{array}{cccc} G_{\theta } & 0 & 0 & G_{\theta s}\\ 0 & G_{\phi } & 0 & G_{\phi s}\\ \Gamma_{\theta } & \Gamma_{\phi } & \Sigma & \Gamma_{s} \end{array}\right]. \end{array}$$

$G_{\theta }=-\mathrm{diag}\left(\left[r_{1}\cos\phi_{1},r_{2}\cos\phi_{2},\ldots ,r_{M}\cos\phi_{M}\right]\right)$, $G_{\phi }=-\mathrm{diag}\left(\left[r_{1},r_{2},\ldots ,r_{M}\right]\right)$, $G_{\theta s}=\mathrm{blkdiag}\left(a_{\theta ,1}^{T},a_{\theta ,2}^{T},\ldots ,a_{\theta ,M}^{T}\right)$, $G_{\phi s}=\mathrm{blkdiag}\left(a_{\phi ,1}^{T},a_{\phi ,2}^{T},\ldots ,a_{\phi ,M}^{T}\right)$, $\Gamma_{\theta }=\left[d_{\theta },H_{\theta }\right]$, $d_{\theta }={\left[r_{2}f_{n,1}^{T}b_{2},r_{3}f_{n,1}^{T}b_{3},\ldots ,r_{M}f_{n,1}^{T}b_{M}\right]}^{T}$, $H_{\theta }=-\mathrm{diag}\left(\left[r_{2}f_{n,2}^{T}b_{1},r_{3}f_{n,3}^{T}b_{1},\ldots ,r_{M}f_{n,M}^{T}b_{1}\right]\right)$, $\Gamma_{\phi }=\left[d_{\phi },H_{\phi }\right]$, $d_{\phi }={\left[r_{2}f_{w,1}^{T}b_{2},r_{3}f_{w,1}^{T}b_{3},\ldots ,r_{M}f_{w,1}^{T}b_{M}\right]}^{T}$, $H_{\phi }=-\mathrm{diag}\left(\left[r_{2}f_{w,2}^{T}b_{1},r_{3}f_{w,3}^{T}b_{1},\ldots ,r_{M}f_{w,M}^{T}b_{1}\right]\right)$, $\Sigma =-\mathrm{diag}(\left[r_{1}{\left(b_{2}-b_{1}\right)}^{T}b_{1},r_{1}{\left(b_{3}-b_{1}\right)}^{T}b_{1},\ldots ,r_{1}{\left(b_{M}-b_{1}\right)}^{T}b_{1}\right])$, $\Gamma_{s}=\left[d_{s},H_{s}\right]$, $d_{s}={\left[g_{21}\left(b_{2}-b_{1}\right),g_{31}\left(b_{3}-b_{1}\right),\ldots ,g_{M1}\left(b_{M}-b_{1}\right)\right]}^{T}$ and $H_{s}=\mathrm{blkdiag}\left(f_{s,2}^{T},f_{s,3}^{T},\ldots ,f_{s,M}^{T}\right)$. The abbreviations diag(·) and blkdiag(·) denote diagonal and block diagonal operations respectively.

Note from model (7) that Gη is zero mean Gaussian noise and the covariance matrix of Gη is $GQG^{T}$. Then, a weighted least-squares (WLS) (The WLS algorithm was presented at the 36th Chinese Control Conference, Dalian, China, July 2017.) estimate of p can be obtained from Equation (21)

(23) $$\begin{array}{c} {\hat{p}}_{\mathrm{WLS}}={\left({\tilde{A}}^{T}W\tilde{A}\right)}^{-1}{\tilde{A}}^{T}W\tilde{h}, \end{array}$$

where $W={\left(GQG^{T}\right)}^{-1}$ is the weighting matrix. To implement the WLS estimator, we need to calculate the weighting matrix W. However, the matrix W is unknown since it depends on the true source position. We can first replace W by an identity matrix to get the least-squares (LS) initial location estimate, and then compute W using the initial position guess and the noisy measurements. Since the performance of WLS estimator is no sensitive to the errors in the weighting matrix W, it does not require an accurate value. Therefore, W is updated after new measurements arrived and only one or two repetitions are enough.

## 4. CLS Estimator Using Joint AOA-GROA Measurements

Note that ${\hat{p}}_{\mathrm{WLS}}$ is biased because the error for $\tilde{h}$ is statistically dependent on the matrix of $\tilde{A}$. The bias of ${\hat{p}}_{\mathrm{WLS}}$ does not vanish as the number of sensors goes to infinity. Inspired by the biased reduced method proposed by Wang and Ho [26], we develop a hybrid constrained least-squares (CLS) method in this section by using the AOA and GROA measurements jointly to reduce the bias.

### 4.1. Constrained Least-Squares Solution

Consider the WLS cost function from Equation (21)

(24) $$\begin{array}{c} J=\xi^{T}{\tilde{D}}^{T}W\tilde{D}\xi , \end{array}$$

where $\tilde{D}=\left[-\tilde{A},\tilde{h}\right]$ and $\xi =\alpha {\left[p^{T},1\right]}^{T}$, and α is a scaling constant. $\tilde{D}$ suffers from additive noise and it can be decomposed as $\tilde{D}=D+\Delta D$, where D is a noiseless version of $\tilde{D}$ and ΔD is the error matrix of $\tilde{D}$. Substituting $\tilde{D}=D+\Delta D$ into Equation (24) yields

(25) $$\begin{array}{c} J=\xi^{T}D^{T}WD\xi +\xi^{T}\Delta D^{T}W\Delta D\xi +2\xi^{T}\Delta D^{T}WD\xi . \end{array}$$

By doing expectation of Formula (25), the last term of Equation (25) is zero and it becomes

(26) $$\begin{array}{c} E\left[J\right]=\xi^{T}D^{T}WD\xi +\xi^{T}E\left[\Delta D^{T}W\Delta D\right]\xi . \end{array}$$

Following from Equation (26), we observe that E[J] attains the minimum if $\xi^{T}E\left[\Delta D^{T}W\Delta D\right]\xi$ is zero. Thus, the constrained least-squares (CLS) solution of cost function (24) can be formulated as

(27) $$\begin{array}{c} \min\xi^{T}{\tilde{D}}^{T}W\tilde{D}\xi \;s.t.\;\xi^{T}\Lambda \xi =c, \end{array}$$

where $\Lambda =E[\Delta D^{T}W\Delta D]$ is the constrained matrix and *c* represents a constant and it can be any value. The above constrained minimization problem can be solved by the Lagrange multiplier approach by constructing the following auxiliary function

(28) $$\begin{array}{c} \xi^{T}{\tilde{D}}^{T}W\tilde{D}\xi +\lambda \left(c-\xi^{T}\Lambda \xi \right), \end{array}$$

where λ is the Lagrange multiplier. The solution of ξ is the generalized eigenvector of the pair $\left({\tilde{D}}^{T}W\tilde{D},\Lambda \right)$ by taking partial derivative of the auxiliary function with respect to ξ, resulting in ${\tilde{D}}^{T}W\tilde{D}\xi =\lambda \Lambda \xi$. Finally, the CLS estimate for source localization is

(29) $$\begin{array}{c} {\hat{p}}_{\mathrm{CLS}}=\frac{\xi (1:3)}{\xi (4)}. \end{array}$$

The use of generalized eigenvector solution to problem (27) was derived in [22] for bearing-only target motion analysis and later generalized to the 3D case [26]. This method requires the constrained matrix Λ to be exactly known and therefore we will discuss how to calculate Λ in the following part.

First, we evaluate the error of the augmented matrix $\tilde{D}$. Using the approximation (15), ΔD is given by

(30) $$\begin{array}{c} \Delta D=RU, \end{array}$$

where R is the (3M−1)×(6M−3) error matrix and elements of R are i.i.d. Gaussian random variables. U is the (6M−3)×4 matrix of the coefficients related to the noise terms. R is given by

(31) $$\begin{array}{c} R=\left[\begin{array}{cccccccccc} R_{n} & R_{s} & 0 & 0 & 0 & 0 & 0 & 0 & 0 & 0\\ 0 & 0 & R_{n} & R_{w} & R_{s} & 0 & 0 & 0 & 0 & 0\\ 0 & 0 & 0 & 0 & 0 & R_{n}^{\prime } & R_{w}^{\prime } & R_{e} & R_{s}^{\prime } & R_{s1} \end{array}\right], \end{array}$$

where $R_{n}=\mathrm{diag}\left(n\right)$, $R_{w}=\mathrm{diag}\left(w\right)$, $R_{e}=\mathrm{diag}\left(e\right)$, $R_{s}=\mathrm{diag}\left(\Delta s\right)$, $R_{n}^{\prime }=\mathrm{diag}\left(\left[n_{2},n_{3},\ldots ,n_{M}\right]\right)$, $R_{w}^{\prime }=\mathrm{diag}\left(\left[w_{2},w_{3},\ldots ,w_{M}\right]\right)$$R_{s}^{\prime }=\mathrm{diag}\left(\left[w_{2},w_{3},\ldots ,w_{M}\right]\right)$ and $R_{s_{1}}=I_{\left(M-1)\times (M-1\right)}\Delta s_{1}$. The matrix U can be written as

(32) $$\begin{array}{c} U={\left[\begin{array}{cccccccccc} \Psi_{\theta n}^{T} & 0 & \Psi_{\phi n}^{T} & \Psi_{\phi w}^{T} & 0 & \Psi_{gn}^{T} & \Psi_{gw}^{T} & \Psi_{ge}^{T} & \Psi_{gs}^{T} & 0\\ \beta_{\theta n}^{T} & \beta_{\theta s}^{T} & \beta_{\phi n}^{T} & \beta_{\phi w}^{T} & \beta_{\phi s}^{T} & \beta_{gn}^{T} & \beta_{gw}^{T} & \beta_{ge}^{T} & \beta_{gs}^{T} & \beta_{gs1}^{T} \end{array}\right]}^{T}, \end{array}$$

where $\Psi_{\theta n}$, $\Psi_{\phi n}$ and $\Psi_{\phi w}$ are the matrices with their *i*th row, i=1,2,…,M given by $\psi_{\theta n,i}={\left[-\cos\theta_{i},-\sin\theta_{i},0\right]}^{T}$, $\psi_{\phi n,i}={\left[\sin\theta_{i}\sin\phi_{i},-\cos\theta_{i}\sin\phi_{i},0\right]}^{T}$ and $\psi_{\phi w,i}={\left[-\cos\theta_{i}\cos\phi_{i},-\sin\theta_{i}\cos\phi_{i},-\sin\phi_{i}\right]}^{T}$. The matrices $\Psi_{gn}$, $\Psi_{gw}$, $\Psi_{ge}$ and $\Psi_{gs}$ are defined with their *j*th row, j=2,3,…,M given by $\psi_{gn,j}=\left(1+g_{j1}\right)\left(f_{n,1}-f_{n,j}\right)$, $\psi_{gw,j}=\left(1+g_{j1}\right)\left(f_{w,1}-f_{w,j}\right)$, $\psi_{ge,j}=b_{1}-b_{j}$ and $\psi_{gs,j}=\frac{1}{r_{1}}\Delta s_{j}^{T}b_{j}\left(b_{1}-b_{j}\right)$. The vectors $\beta_{\theta n}$, $\beta_{\phi n}$, $\beta_{\phi w}$, $\beta_{gn}$, $\beta_{gw}$ and $\beta_{ge}$ are given by $\beta_{\theta n}=-{\left[\psi_{\theta n,1}^{T}s_{1},\psi_{\theta n,2}^{T}s_{2},\ldots ,\psi_{\theta n,M}^{T}s_{M}\right]}^{T}$, $\beta_{\theta s}=-{\left[a_{\theta ,1}^{T},a_{\theta ,2}^{T},\ldots ,a_{\theta ,M}^{T}\right]}^{T}$, $\beta_{\phi n}=-{\left[\psi_{\phi n,1}^{T}s_{1},\psi_{\phi n,2}^{T}s_{2},\ldots ,\psi_{\phi n,M}^{T}s_{M}\right]}^{T}$, $\beta_{\phi w}=-{\left[\psi_{\phi w,1}^{T}s_{1},\psi_{\phi w,2}^{T}s_{2},\ldots ,\psi_{\phi w,M}^{T}s_{M}\right]}^{T}$, $\beta_{\phi s}=-{\left[a_{\phi ,1}^{T},a_{\phi ,2}^{T},\ldots ,a_{\phi ,M}^{T}\right]}^{T}$, $\beta_{gn}={\left[\left(f_{n,2}^{T}-f_{n,1}^{T}\right)s_{2},\ldots ,\left(f_{n,M}^{T}-f_{n,1}^{T}\right)s_{M}\right]}^{T}$, $\beta_{gw}={\left[\left(f_{w,2}^{T}-f_{w,1}^{T}\right)s_{2},\ldots ,\left(f_{w,M}^{T}-f_{w,1}^{T}\right)s_{M}\right]}^{T}$, $\beta_{ge}={\left[{\left(b_{2}-b_{1}\right)}^{T}s_{1},{\left(b_{3}-b_{1}\right)}^{T}s_{1},\ldots ,{\left(b_{M}-b_{1}\right)}^{T}s_{1}\right]}^{T}$, $\beta_{gs}={\left[{\left(f_{s,2}^{\prime }\right)}^{T},{\left(f_{s,3}^{\prime }\right)}^{T},\ldots ,{\left(f_{s,M}^{\prime }\right)}^{T}\right]}^{T}$, $f_{s,j}^{\prime }=\left(b_{j}-b_{1}\right)+\frac{1}{r_{1}}s_{1}^{T}\left(b_{j}-b_{1}\right)b_{j}$, and $\beta_{gs1}={\left[g_{21}{\left(b_{2}-b_{1}\right)}^{T},g_{31}{\left(b_{3}-b_{1}\right)}^{T},\ldots ,g_{M1}{\left(b_{M}-b_{1}\right)}^{T}\right]}^{T}$. As long as ΔD is obtained, the constrained matrix is equal to

(33) $$\begin{array}{c} \Lambda =E\left[\Delta D^{T}W\Delta D\right]=U^{T}E\left[R^{T}WR\right]U, \end{array}$$

where $E[R^{T}WR]$ is given in Appendix A.

### 4.2. Performance Analysis

To illustrate the performance of CLS estimator, we need to develop the theoretical MSE. We first compute the CRLB of the proposed estimator and show that the theoretical MSE of the CLS estimator is equal to the CRLB over the small error region. The CRLB provides a benchmark for the performance comparison of any unbiased estimator. Let $\vartheta ={\left[p^{T},s^{T}\right]}^{T}.$ The CRLB matrix of the parameter vector ϑ is given by [34]

(34) $$\begin{array}{c} \mathrm{CRLB}\left(\vartheta \right)={\mathrm{FIM}}^{-1}\left(\vartheta \right)={\left[\begin{array}{cc} \frac{\partial \kappa^{T}}{\partial p}Q_{\kappa }^{-1}\frac{\partial \kappa }{\partial p^{T}} & \frac{\partial \kappa^{T}}{\partial p}Q_{\kappa }^{-1}\frac{\partial \kappa }{\partial s^{T}}\\ \frac{\partial \kappa^{T}}{\partial s}Q_{\kappa }^{-1}\frac{\partial \kappa }{\partial p^{T}} & \frac{\partial \kappa^{T}}{\partial s}Q_{\kappa }^{-1}\frac{\partial \kappa }{\partial s^{T}}+Q_{s}^{-1} \end{array}\right]}^{-1}, \end{array}$$

where FIM represents the Fisher information matrix, $\kappa ={\left[\theta^{T},\phi^{T},g^{T}\right]}^{T}$, $\frac{\partial \kappa }{\partial p^{T}}={\left[L_{\theta }^{T},L_{\phi }^{T},q^{T}\right]}^{T}$ and $\frac{\partial \kappa }{\partial s^{T}}=-{\left[L_{\theta }^{T},L_{\phi }^{T},q^{T}\right]}^{T}$. The matrices $L_{\theta }$ and $L_{\phi }$ are defined with their *i*th row, i=1,2,…,M given as

(35) $$\begin{array}{cc} l_{\theta ,i}^{T} & =\frac{1}{r_{i}}\left[-\sin\theta_{i}/\cos\phi_{i},\cos\theta_{i}/\cos\phi_{i},0\right], \end{array}$$

(36) $$\begin{array}{cc} l_{\phi ,i}^{T} & =\frac{1}{r_{i}}\left[-\cos\theta_{i}\sin\phi_{i},-\sin\theta_{i}\sin\phi_{i},\cos\phi_{i}\right]. \end{array}$$

The vector q is defined by

(37) $$\begin{array}{c} q={\left[\frac{b_{2}}{r_{1}}-\frac{r_{2}b_{1}}{r_{1}^{2}},\frac{b_{3}}{r_{1}}-\frac{r_{3}b_{1}}{r_{1}^{2}},\ldots ,\frac{b_{M}}{r_{1}}-\frac{r_{M}b_{1}}{r_{1}^{2}}\right]}^{T}. \end{array}$$

According to the block matrix inversion formula [34], we have the inverse of CRLB matrix with respect to p

(38) $$\begin{array}{c} \mathrm{CRLB}{\left(p\right)}^{-1}=\frac{\partial \kappa^{T}}{\partial p}Q_{\kappa }^{-1}\frac{\partial \kappa }{\partial p^{T}}-\frac{\partial \kappa^{T}}{\partial p}Q_{\kappa }^{-1}\frac{\partial \kappa }{\partial s^{T}}\left(\frac{\partial \kappa^{T}}{\partial p}Q_{\kappa }^{-1}\frac{\partial \kappa }{\partial p^{T}}+Q_{s}^{-1}\right)\frac{\partial \kappa^{T}}{\partial s}Q_{\kappa }^{-1}\frac{\partial \kappa }{\partial p^{T}}. \end{array}$$

It is demonstrated that the inverse of theoretical MSE matrix for CLS estimator is [26]

(39) $$\begin{array}{c} \mathrm{COV}{\left({\hat{p}}_{\mathrm{CLS}}\right)}^{-1}\approx A^{T}WA=A^{T}{\left(GQG^{T}\right)}^{-1}A. \end{array}$$

We let $G=[G_{\kappa },G_{s}]$, where

(40) $$\begin{array}{c} G_{\kappa }=\left[\begin{array}{ccc} G_{\theta } & 0 & 0\\ 0 & G_{\phi } & 0\\ \Gamma_{\theta } & \Gamma_{\phi } & \Sigma \end{array}\right],\;G_{s}=\left[\begin{array}{c} G_{\theta s}\\ G_{\phi s}\\ \Gamma_{s} \end{array}\right]. \end{array}$$

Substituting Equation (40) into Equation (39) and applying the matrix inversion lemma [34] on Equation (39) leads to

(41) $$\begin{array}{cc} \mathrm{COV}{\left({\hat{p}}_{\mathrm{CLS}}\right)}^{-1} & ={\left(G_{\kappa }^{-1}A\right)}^{T}Q_{\kappa }^{-1}G_{\kappa }^{-1}A\\ & -{\left(G_{\kappa }^{-1}A\right)}^{T}Q_{\kappa }^{-1}G_{\kappa }^{-1}G_{s}{\left(Q_{s}^{-1}+{\left(G_{\kappa }^{-1}G_{s}\right)}^{T}Q_{\kappa }^{-1}G_{\kappa }^{-1}G_{s}\right)}^{-1}{\left(G_{\kappa }^{-1}G_{s}\right)}^{T}Q_{\kappa }^{-1}G_{\kappa }^{-1}A. \end{array}$$

We are interested in finding $\mathrm{COV}\left({\hat{p}}_{\mathrm{CLS}}\right)=\mathrm{CRLB}\left(p\right)$. From the expressions of $G_{\theta }$,$G_{\phi }$, $L_{\theta }$ and $L_{\phi }$, we first obtain

(42) $$\begin{array}{c} G_{\theta }L_{\theta }=A_{\theta },\;G_{\phi }L_{\phi }=A_{\phi }. \end{array}$$

Then, using the matrices $\Gamma_{\theta }$, $\Gamma_{\phi }$, Σ and $\frac{\partial \kappa }{\partial p^{T}}$, we have

(43) $$\begin{array}{c} v_{j}^{T}=-{\left(b_{j}-b_{1}\right)}^{T}r_{1}b_{1}\left(\frac{b_{j}}{r_{1}}-\frac{r_{j}b_{1}}{r_{1}^{2}}\right)+r_{j}f_{n,1}^{T}b_{j}l_{\theta ,1}^{T}+r_{j}f_{w,1}^{T}b_{j}l_{\phi ,1}^{T}-r_{j}f_{n,j}^{T}b_{1}l_{\theta ,j}^{T}-r_{j}f_{w,j}^{T}b_{1}l_{\phi ,j}^{T} \end{array}$$

for j=2,3,…,M. Combining Equations (42) and (43), we observe that $\frac{\partial \kappa }{\partial p^{T}}=G_{\kappa }^{-1}A$. Similarly, we have $\frac{\partial \kappa }{\partial s^{T}}=-G_{\kappa }^{-1}G_{s}$. Substituting these two expressions into Equation (38) results in $\mathrm{CRLB}\left(p\right)=\mathrm{COV}\left({\hat{p}}_{\mathrm{CLS}}\right)$.

## 5. Simulation Results

In this section, we illustrate the performance of the proposed CLS method using AOA and GROA measurements jointly and compare it with the bearing-only WLS and CLS algorithms. We also include the WLS estimator from (23). The weighted matrix utilized in the WLS algorithm is calculated according to the result of the LS method.

**Figure 2:**
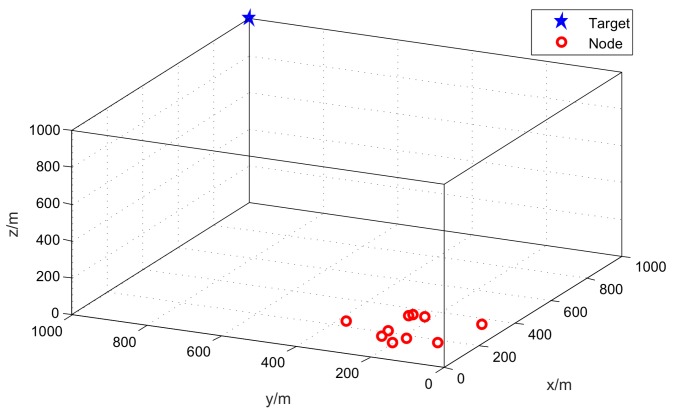
The geometry of the target and nodes.

In the simulations, we assume that *M* acoustic nodes are placed on the unmanned aerial vehicles (UAVs). The UAVs are networked together to localize an aeroplane or a helicopter. The UAVs are flying at the same speed and in the same direction. The positions of UAVs are generated randomly within a 500 m × 500 m × 50 m cube. The target location is set at (1000 m, 1000 m, 1000 m). Figure 2 plots the sensor-target geometry, where the red circle denotes the position of the node and the blue star represents the location of the target.

The acoustic nodes on each UAV platform can take azimuth, elevation and GROA measurements simultaneously. The azimuth error, elevation error, GROA error and the sensor position error are independent and they follow zero mean Gaussian distribution with diagonal covariance matrices $Q_{n}$, $Q_{w}$, $Q_{e}$ and $Q_{s}$. For simplicity, the covariance matrices of azimuth noise and elevation noise are set equal and $Q_{n}=Q_{w}=\sigma_{AOA}^{2}I$. We make comparisons over various AOA, GROA and sensor position errors, whose noise powers are denoted by $\sigma_{AOA}^{2}$, $\sigma_{GROA}^{2}$ and $\sigma_{s}^{2},$ respectively. We will examine the localization accuracy where one noise variance varies while the other noise powers are fixed. To alleviate the dependency of a particular geometry, we first do experiments over 100 random geometries. For each geometry, the number of Monte Carlo runs is 50.

### 5.1. Fixed Number of Nodes

We first consider the source localization scenario when the number of nodes is fixed at 10. To compare the performance of the algorithms, we compute the average mean square error (*MSE*),

(44) $$\begin{array}{c} MSE=\frac{1}{K}\sum_{k=1}^{K}\left[{\left(x-{\hat{x}}_{k}\right)}^{2}+{\left(y-{\hat{y}}_{k}\right)}^{2}+{\left(z-{\hat{z}}_{k}\right)}^{2}\right], \end{array}$$

where $\left({\hat{x}}_{k},{\hat{y}}_{k},{\hat{z}}_{k}\right)$ is the estimated target position at the *k*-th simulation run, and *K* is the total number of runs. In the figures drawn as follows, we will use log-scale for MSE to show the wide range of the levels examined in this example, which is given by 10lg(MSE).

We set the standard deviation of the AOA measurement noise from 0.5° (0.0087 radian) to 5° (0.087 radian), that of the GROA noise from 10^−4^ to 10^−2^ and that of the sensor position error from 10^−2^ m to 10 m.

**Figure 3:**
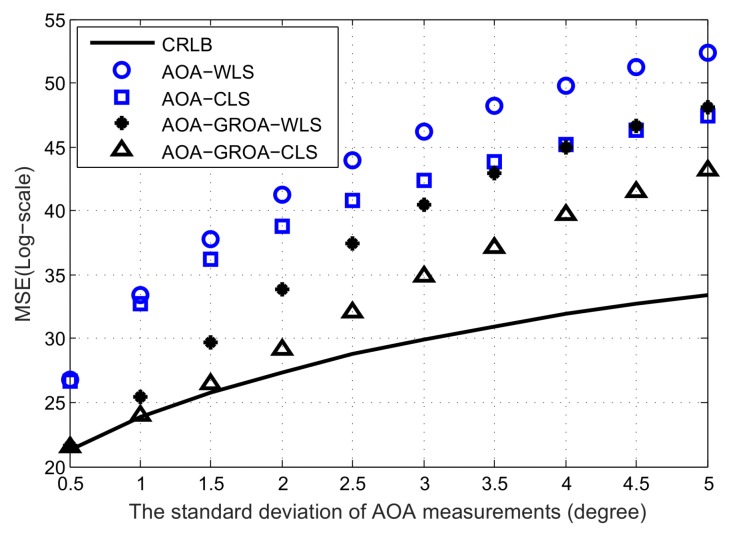
Average MSE results of AOA-only WLS, AOA-only CLS, AOA-GROA WLS and AOA-GROA CLS methods, $\sigma_{AOA}$ varies, whereas $\sigma_{GROA}$ = 3 × 10^−3^ and $\sigma_{s}$ = 10^−2^ m.

In Figure 3, we plot the average MSE results as $\sigma_{AOA}$ varies, where $\sigma_{GROA}$ and $\sigma_{s}$ are fixed at 3 × 10^−3^ and 10^−2^ m, respectively. The MSEs of the AOA-only WLS, AOA-only WLS, AOA-GROA WLS and AOA-GROA CLS methods are drawn by the blue circle, blue square, black star and black upper triangle, respectively. The black solid line is the CRLB of the proposed method. As can be seen, the proposed CLS estimator using AOA-GROA outperforms the other methods, and it can achieve CRLB when $\sigma_{AOA}$ is below 1.5°. From Figure 3, we observe that the GROA information can be used to improve location accuracy. The AOA-GROA WLS algorithm outperforms the AOA-WLS method to 8 dB. Note that a difference of 3 dB is equivalent to multiplying 2 in the MSE calculation. Compared to the AOA-CLS method, the AOA-GROA CLS algorithm has better performance and the improvement is about 9 dB. The performance of the AOA-GROA WLS and the AOA-GROA CLS are quite close when $\sigma_{AOA}$ is below 0.5°. This is because the biases of both methods are small at a low noise level. However, the AOA-GROA CLS is more beneficial to reducing the bias when $\sigma_{AOA}$ is above 1°.

**Figure 4:**
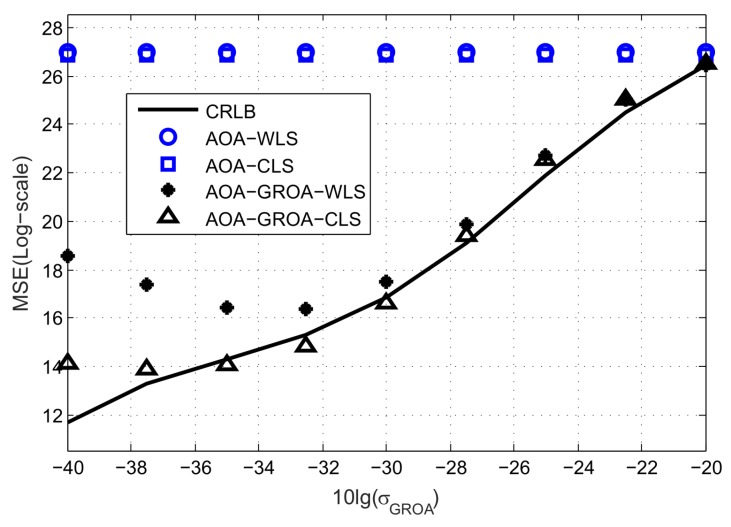
Average MSE results of AOA-only WLS, AOA-only CLS, AOA-GROA WLS and AOA-GROA CLS methods, $\sigma_{GROA}$ varies, whereas $\sigma_{AOA}$ = 10^−2^ radian and $\sigma_{s}$ = 10^−2^ m.

Figure 4 shows the average MSEs of the methods as $\sigma_{GROA}$ varies, where $\sigma_{AOA}$ and $\sigma_{s}$ are fixed at 10^−2^ radian and 10^−2^ m. When $\sigma_{GROA}$ is above 10^−2^, using both AOAs and GROAs gives about the same accuracy as using AOAs only. This is because the proposed algorithm puts much more weight on AOAs rather than GROAs at a high level of $\sigma_{GROA}$. When $\sigma_{GROA}$ is below 5 × 10^−4^, the average MSE of the proposed method does not reduce as the value of $\sigma_{GROA}$ gets small. This is because the performance is dominated by the GROAs and the bias introduced by the GROAs can not be neglected. The estimation results of the AOA-only WLS and the AOA-only CLS in Figure 4 are the same because these two methods are not related to $\sigma_{GROA}$.

**Figure 5:**
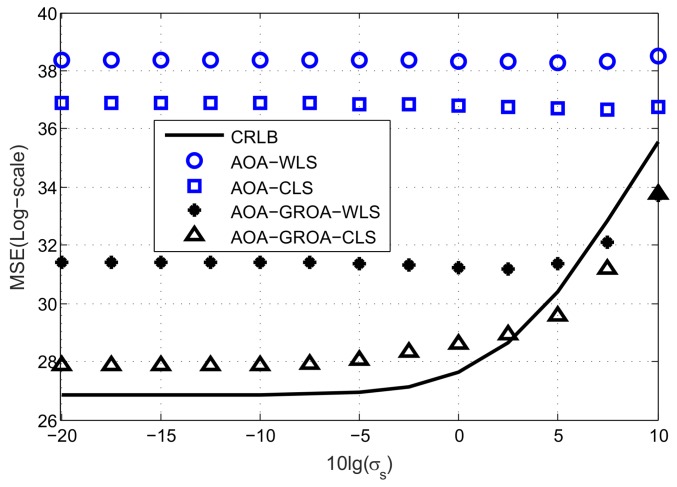
Average MSE results of AOA-only WLS, AOA-only CLS, AOA-GROA WLS and AOA-GROA CLS methods, $\sigma_{s}$ varies, whereas $\sigma_{AOA}$ = 10^−2^ radian and $\sigma_{GROA}$ = 3 × 10^−3^.

Figure 5 depicts the average MSE performance of the methods as $\sigma_{s}$ varies, where $\sigma_{AOA}$ and $\sigma_{GROA}$ are fixed at 10^−2^ radian and 3 × 10^−3^, respectively. Figure 5 validates the proposed AOA-GROA CLS method, which provides superior performance on the sensor position uncertainty. The AOA-GROA CLS method is very useful for a small value of $\sigma_{s}$. When $\sigma_{s}$ reaches 10 m, the results of using the AOA-GROA WLS and AOA-GROA CLS methods are almost the same.

### 5.2. Changed Number of Nodes

In this subsection, we consider the location accuracy of various methods when the number of nodes varies. The number of nodes changes from 3 to 100. All nodes are randomly placed within a 500 m × 500 m × 50 m cube. The position of the target is fixed at (1000, 1000, 1000) m. As such, we can illustrate that the number of nodes can affect the location accuracy.

**Figure 6:**
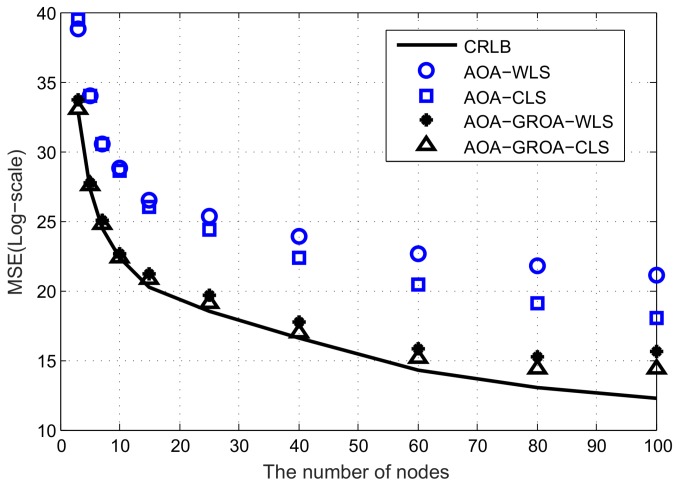
Average MSE results of AOA-only WLS, AOA-only CLS, AOA-GROA WLS and AOA-GROA CLS methods with respect to various number of nodes, $\sigma_{AOA}$ = 0.01 radian, $\sigma_{GROA}$ = 3 × 10^−3^ and $\sigma_{s}$ = 10^−2^.

**Figure 7:**
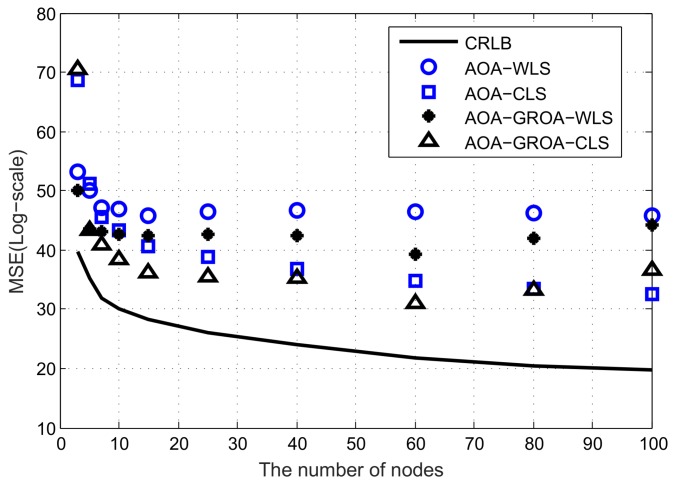
Average *MSE* results of AOA-only WLS, AOA-only CLS, AOA-GROA WLS and AOA-GROA CLS methods with respect to various number of nodes, $\sigma_{AOA}$ = 0.05 radian, $\sigma_{GROA}$ = 3 × 10^−3^ and $\sigma_{s}$ = 10^−2^.

Similar to the experiments done in the case of fixed number of nodes, we do comparisons with a various number of nodes using the localization methods mentioned above. All results are shown in Figure 6 and Figure 7. Figure 6 compares the MSEs of the solutions, where the standard deviations of $\sigma_{AOA}$, $\sigma_{GROA}$ and $\sigma_{s}$ are fixed at 10^−2^ radian, 3 × 10^−3^ and 10^−2^ m, respectively. Figure 7 illustrates the *MSE* performance, where the standard deviations of $\sigma_{AOA}$, $\sigma_{GROA}$ and $\sigma_{s}$ are fixed at 0.05 radian, 3 × 10^−3^ and 10^−2^ m, respectively.

In these two figures, the localization algorithms appear to have better performance as the number of nodes increases. Note that the localization methods can significantly decrease *MSE* when the number of nodes is below 15. On the contrary, the localization algorithms improve performance very slowly when the number of nodes is above 20. A large number of nodes do not provide much benefit. For practical applications, we need to balance the costs of devices against their localization accuracy. If the localization accuracy is designated in advance, node selection is required to optimize the lifetime of ASNs and this will be our future work.

## 6. Conclusions

In this paper, we propose a CLS source localization method for acoustic sensor networks by jointly using the AOA and GROA measurements in the presence of node position errors. The proposed AOA-GROA CLS estimator is simple to implement and it does not require the initial guess. Compared to the AOA-only method, the proposed algorithm has better performance with the assistance of additional GROA information. Simulations validate the performance of the proposed estimator. The theoretical performance analysis is also conducted in this paper, and it predicts that the MSE of the proposed method can attain the CRLB over the small Gaussian region.

## Abbreviations

The following abbreviations are used in this manuscript:

AOA	Angle of arrival
ASN	Acoustic sensor network
BOPLE	Bearing-only pseudolinear estimator
CLS	Constrained least-squares
CRLB	Cramér–Rao lower bound
GROA	Gain ratio of arrival
LS	Least-squares
ML	Maximum likelihood
MSE	Mean square error
TDOA	Time difference of arrival
WIV	Weight instrumental variables
WLS	Weighted least-squares

## Appendix A

Assume that the noise vectors n, w and e are independent from each other and the measurement noise from each sensor is subject to independence. Then, the covariance matrix of η can be written as

(A1) $$\begin{array}{c} Q=\left[\begin{array}{cccc} Q_{n} & 0 & 0 & 0\\ 0 & Q_{w} & 0 & 0\\ 0 & 0 & Q_{e} & 0\\ 0 & 0 & 0 & Q_{s} \end{array}\right]. \end{array}$$

Note that Q is a diagonal matrix and the weight matrix $W={\left(G^{T}QG\right)}^{-1}$ is symmetrical. If we partition the weighting matrix W as follows:

(A2) $$\begin{array}{c} W=\left[\begin{array}{ccc} W_{11} & W_{12} & W_{13}\\ W_{12} & W_{22} & W_{23}\\ W_{13}^{T} & W_{23}^{T} & W_{33} \end{array}\right], \end{array}$$

where $W_{11}$, $W_{22}$ and $W_{12}$ have the same size M×M, the matrices $W_{13}$, $W_{23}$ have size M×(M−1) and $W_{33}$ is a (M−1)×(M−1) matrix. According to (26), it is straightforward to have

(A3) $$\begin{array}{c} E\left[R^{T}WR\right]=\left[\begin{array}{cccccccccc} Q_{n}^{11} & 0 & Q_{n}^{12} & 0 & 0 & {\bar{Q}}_{n}^{13} & 0 & 0 & 0 & 0\\ 0 & Q_{s}^{11} & 0 & 0 & Q_{s}^{12} & 0 & 0 & 0 & {\bar{Q}}_{s}^{13} & {\bar{Q}}_{s1}^{13}\\ Q_{n}^{12} & 0 & Q_{n}^{22} & 0 & 0 & {\bar{Q}}_{n}^{23} & 0 & 0 & 0 & 0\\ 0 & 0 & 0 & Q_{w}^{22} & 0 & 0 & {\bar{Q}}_{w}^{23} & 0 & 0 & 0\\ 0 & Q_{s}^{12} & 0 & 0 & Q_{s}^{22} & 0 & 0 & 0 & {\bar{Q}}_{s}^{23} & {\bar{Q}}_{s1}^{23}\\ {\left({\bar{Q}}_{n}^{13}\right)}^{T} & 0 & {\left({\bar{Q}}_{n}^{23}\right)}^{T} & 0 & 0 & {\bar{Q}}_{n}^{33} & 0 & 0 & 0 & 0\\ 0 & 0 & 0 & {\left({\bar{Q}}_{w}^{23}\right)}^{T} & 0 & 0 & {\bar{Q}}_{w}^{33} & 0 & 0 & 0\\ 0 & 0 & 0 & 0 & 0 & 0 & 0 & Q_{e}^{33} & 0 & 0\\ 0 & {\left({\bar{Q}}_{s}^{13}\right)}^{T} & 0 & 0 & {\left({\bar{Q}}_{s}^{23}\right)}^{T} & 0 & 0 & 0 & {\bar{Q}}_{s}^{33} & 0\\ 0 & {\left({\bar{Q}}_{s1}^{13}\right)}^{T} & 0 & 0 & {\left({\bar{Q}}_{s1}^{23}\right)}^{T} & 0 & 0 & 0 & 0 & {\bar{Q}}_{s1}^{33} \end{array}\right], \end{array}$$

where $Q_{n}^{11}=Q_{n}⊙W_{11}$, $Q_{n}^{12}=Q_{n}⊙W_{12}$, $Q_{n}^{22}=Q_{n}⊙W_{22}$, ${\bar{Q}}_{n}^{13}={\left[0_{(M-1)\times 1},Q_{n}^{\prime }\right]}^{T}⊙W_{13}$, $Q_{n}^{\prime }=\mathrm{diag}\left(\left[n_{2},n_{3},\ldots ,n_{M}\right]\right)$, ${\bar{Q}}_{n}^{23}={\left[0_{(M-1)\times 1},Q_{n}^{\prime }\right]}^{T}⊙W_{23}$, ${\bar{Q}}_{n}^{33}=Q_{n}^{\prime }⊙W_{33}$, $Q_{w}^{22}=Q_{w}⊙W_{22}$, ${\bar{Q}}_{w}^{23}={\left[0_{(M-1)\times 1},Q_{w}^{\prime }\right]}^{T}⊙W_{23}$, $Q_{w}^{\prime }=\mathrm{diag}\left(\left[w_{2},w_{3},\ldots ,w_{M}\right]\right)$, ${\bar{Q}}_{w}^{33}=Q_{w}^{\prime }⊙W_{33}$, $Q_{e}^{33}=Q_{e}⊙W_{33}$.
